# Treatment sequence patterns of urate-lowering therapy in Korean patients with gout: A common data model-based study

**DOI:** 10.1371/journal.pone.0347654

**Published:** 2026-04-17

**Authors:** Min Jung Kim, Eun-Gee Park, Changyoung Kim, Dong Yoon Kang, Borim Ryu, Kichul Shin

**Affiliations:** 1 Division of Rheumatology, Department of Internal Medicine, Seoul Metropolitan Government–Seoul National University Boramae Medical Center, Seoul, Republic of Korea; 2 Data Science Center, Biomedical Research Institute, Seoul Metropolitan Government–Seoul National University Hospital Boramae Medical Center, Seoul, Republic of Korea; 3 BigData Center, Ulsan University Hospital, Ulsan, Republic of Korea; 4 Department of Preventive Medicine, Ulsan University Hospital, University of Ulsan College of Medicine, Ulsan, Republic of Korea; 5 Department of Internal Medicine, Seoul National University College of Medicine, Seoul, Republic of Korea; Straub Medical Center, UNITED STATES OF AMERICA

## Abstract

**Background:**

A treat-to-target strategy involving treatment modification improves outcomes in gout, but evidence remains limited regarding the optimal approach when initial urate-lowering therapy (ULT) fails. This study aimed to investigate real-world ULT sequence patterns and evaluate treatment retention based on the initial agent, modification type, and comorbidities.

**Method:**

We analyzed electronic health record data collected from 2010 to 2022 from the common data model databases of two hospitals. Adults aged 18 years or older diagnosed with gout who initiated ULT and were followed for at least 2 years were included. Outcomes included the frequency and sequence of ULT prescriptions. Treatment modification, defined as switching to another ULT or adding an additional agent, was considered the end of retention for the previous regimen. Subgroup analyses were performed based on comorbidity.

**Results:**

Among 2220 patients, febuxostat was the most common first-line agent (51.4%), with 90.9% maintaining therapy. Among those who modified febuxostat therapy, switchers and add-on users continued treatment similarly (91.5% *vs*. 86.8%, *P* = 0.33). Of allopurinol initiators, 55.8% changed therapy, mainly switching to febuxostat or benzbromarone rather than adding another agent (51.4% *vs*. 4.1%, *P* < 0.001), with similar retention rates (91.5% *vs*. 86.8%, *P* = 0.33). Among benzbromarone initiators, 57.2% changed therapy (switchers, 56.7%; add-on users, 0.5%; *P* < 0.001), and retention rates were 90.5% and 100%, respectively (*P* = 1.00). Chronic kidney disease was associated with low variability in ULT sequence.

**Conclusions:**

ULT demonstrated durable retention when used as first- or second-line treatment, with switching being more common than add-on therapy and maintaining similar retention rates.

## Introduction

Urate-lowering therapy (ULT) is the cornerstone of long-term gout management. The main goal of ULT is to maintain serum urate levels below 6 mg/dL (< 0.36 mmol/L), thereby preventing new monosodium urate crystal formation, promoting crystal dissolution, reducing gout flares, and resolving tophi [1]. Current international guidelines recommend a treat-to-target approach, with ULT modification guided by serial serum urate measurements [2–4].

Allopurinol and febuxostat are two effective agents for achieving target serum urate levels in most patients [5]. Allopurinol, a conventional xanthine oxidase inhibitor (XOI), has long been used in general settings, whereas febuxostat is a newer, more selective and potent XOI with a simpler dosing regimen [6]. Uricosuric agents such as probenecid, benzbromarone, and lesinurad enhance renal urate excretion. Despite the limited number of urate-lowering agents, these medications effectively achieve the treatment target, rendering gout a largely controllable condition when appropriately managed.

Nonetheless, some patients fail to achieve target serum urate levels because of insufficient efficacy, adverse events, inadequate dose escalation, or poor adherence [7]. When target serum urate levels are not achieved despite reasonable compliance and appropriate dose titration, treatment modification, such as switching to another agent or adding an additional one, is often required. However, when initial therapy fails, evidence guiding subsequent treatment strategies remains limited. In particular, data on optimal sequencing or combination strategies for ULT are scarce in Asian populations, who have distinct genetic and pharmacological characteristics.

Unlike in Western countries, both allopurinol and febuxostat are widely used as first-line treatments in Korea. Owing to the limited evidence, recent Korean guidelines do not yet specify which agent should be initiated or the preferred treatment sequence when the response to initial therapy is inadequate [8]. Given these gaps, understanding real-world prescription patterns and sequential ULT use could provide important insights into optimizing gout management. The use of electronic health record (EHR) data standardized through a common data model (CDM) enables multicenter real-world analyses across institutions. Using longitudinal CDM data from two hospitals in Korea, we aimed to characterize real-world ULT sequences in patients with gout. Treatment retention was analyzed as a secondary, descriptive measure to provide context to sequencing patterns by initial agent treatment modification type (switch or add-on), and comorbid conditions.

## Materials and methods

### Data sources

This multicenter, retrospective, observational cohort study utilized the Observational Medical Outcomes Partnership CDM version 5.4 of the Observational Health Data Sciences and Informatics (OHDSI) network. EHR data were obtained from two Korean hospitals: Seoul National University Boramae Medical Center (BMC) and Ulsan University Hospital (UUH). BMC is a 786-bed secondary hospital in Seoul with an EHR database of 1033461 patients who visited between 2010 and 2022. UUH is a 1000-bed tertiary hospital in Ulsan, Southeast Korea, providing data from 717922 patients recorded from 2010 to 2022.

This study was conducted in accordance with the principles of the Declaration of Helsinki. The Institutional Review Board (IRB) of both hospitals reviewed the study protocol, which was exempted from full approval and informed consent requirements because all data were de-identified (BMC IRB no.: 07-2023-11, UUH IRB no.: 2024-03-004). We accessed the data between June 2023 and June 2024 and had no access to identifiable information.

### Study population and cohort definitions

Cohorts were defined using ATLAS, an open-source OHDSI platform. The target cohort included patients aged 18 years or older with a diagnosis of gout identified in each hospital’s CDM database. Eligible patients had at least one prescription for a urate-lowering agent (allopurinol, febuxostat, or benzbromarone) available in Korea and at least two years of follow-up data from the index date, defined as the first gout diagnosis. Previous validation studies support the reliability of code-based algorithms for identifying patients with gout. Notably, a Korean claims-based study demonstrated high diagnostic performance for an algorithm that combines gout diagnosis codes with prescriptions for allopurinol or febuxostat and more than one clinic visit in the subsequent year (sensitivity 96.4%, specificity 98.5%) [9], which is conceptually consistent with our cohort definition.

Cohort definitions used international standard terminologies from the OHDSI vocabulary: Systematized Nomenclature of Medicine-Clinical Terms for diagnoses [10,11], RxNorm for drug exposures [11 12], and Logical Observation identifies Names and Codes for laboratory tests, such as serum urate levels [11 13]. The use of these standardized vocabularies ensured consistency across databases. The detailed concept sets used in each cohort definition are provided in S1 Table in S1 File.

### Outcomes

We assessed the frequency and sequence of ULT prescriptions during the study period. Treatment lines were defined by distinct drug exposure eras. The end of one treatment line was defined as the termination of a drug era due to switching to a different ULT agent or initiating an additional ULT agent. In the case of add-on therapy, the original monotherapy was considered to have ended, and a new treatment line representing the combination regimen was initiated. Continued use of the same drug without switching or adding another agent was considered retention within the same line. Dose changes were not included in the definition of treatment modification. Treatment discontinuation without subsequent initiation of another ULT was not modeled as a separate treatment state within the pathway framework. Accordingly, retention was defined as the absence of switching or add-on therapy.

### Statistical analysis and sequential treatment pattern analysis

Baseline characteristics were summarized using descriptive statistics. Categorical variables were compared using the Chi-square or Fisher’s exact test, as appropriate. Sequential treatment patterns were analyzed using 1) the Cohort Pathways function in ATLAS, and 2) the open-source R package *TreatmentPatterns* for sensitivity analysis [14 15]. For patients who switched drugs and later returned to the original one, only their initial exposure was recorded. Analysis settings were as follows: “collapse days or combination window” = 0, “minimum cell count” = 5, and “maximum path length” = 5 for Cohort Pathways; eraCollapseSize = 0 and miniPostCombinationDuration = 0 for *TreatmentPatterns* [14]. Analyses were independently conducted at each hospital, and the counts of each sequence were combined across database. Sunburst plots were generated to visualize treatment sequences, with the innermost ring representing first-line therapy and subsequent rings showing later lines. Baseline comorbidities were extracted using the R package *FeatureExtraction*, which identifies conditions recorded during the 365 days prior to and including the index date. Subgroup analyses focused on comorbid chronic kidney disease (CKD) or end-stage renal disease (ESRD), defined using concept IDs in ATLAS (S1 Table in S1 File). Sensitivity analyses were performed for 1) patients continuously exposed to ULT every 120 days after the index date, and 2) those whose serum urate remained ≥ 6 mg/dL between 6 and 12 months after the index date, representing possible treatment non-response rather than intolerance. All statistical tests were two-sided, and *P* < 0.05 was considered statistically significant. Analyses were performed using R version 4.2.2 (R Foundation for Statistical Computing, Vienna, Austria).

## Results

### Patient characteristics

In total, 2743 patients with gout were identified from the two CDM databases: 1317 from BMC and 1426 from UUH. Baseline demographic and clinical characteristics are summarized in Table 1. Most patients were male, and the majority were between 50 and 70 years of age. The most common comorbidities were hypertension, renal impairment, hyperlipidemia, and diabetes (Table 1). Using the Cohort Pathways function in ATLAS, a total of 2232 patients (1019 from BMC and 1213 from UUH) were included in the final treatment sequence analysis. Patients who received ULT only before, but not after, the index gout diagnosis were excluded.

**Table 1:** Baseline characteristics of the study population.

Characteristics	BMC (n = 1317)	UUH (n = 1426)
Age group, years (%)		
18 - 19	0.2	0.2
20 - 29	3.4	3.4
30 - 39	10.6	12.0
40 - 49	12.9	18.1
50 - 59	20.1	28.4
60 - 69	21.7	22.5
70 - 79	23.0	13.1
80 -	8.3	2.5
Male (%)	90.4	92.6
Medical history (%)		
Chronic liver disease	1.4	1.1
Chronic obstructive lung disease	2	1.2
Diabetes mellitus	11.6	8.4
Hyperlipidemia	14.8	5.2
Hypertensive disorder	23.5	13.5
Renal impairment	14.4	13
Atrial fibrillation	5	4.6
Cerebrovascular disease	4.8	1.3
Heart failure	4.3	2.9
Ischemic heart disease	4.8	6.3
Malignant neoplasm	5.2	5.4

BMC, Boramae Medical Center; UUH, Ulsan University Hospital.

### Sequential treatment patterns of ULT

**Fig 1:**
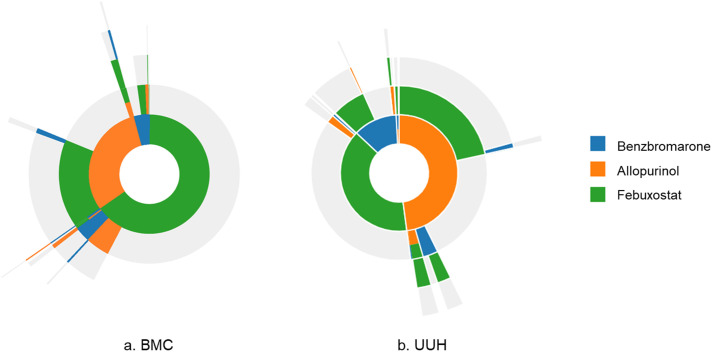
Sunburst plot showing treatment pathways of urate-lowering therapy in gout patients from each database. **(a)** Boramae Medical Center (BMC) and **(b)** Ulsan University Hospital (UUH).

Fig 1 and Table 2 summarize treatment sequences at BMC and UUH. Overall, febuxostat was the most commonly prescribed initial ULT (51.4%), followed by allopurinol (40.2%) and benzbromarone (8.4%). Initial combination therapy was rare (< 1%), and treatment patterns were largely consistent across the two institutions. Among febuxostat initiators, the vast majority continued therapy without modification, with only a small proportion switching or receiving add-on therapy. In contrast, approximately half of allopurinol and benzbromarone initiators modified their initial treatment, predominantly by switching rather than by add-on therapy (Table 2). The detailed treatment sequences are presented in S6 Table in S1 File.

**Table 2:** Sequential treatment pattern of urate-lowering therapy.

Initial therapy^a^	Cohort	n	Retention, n (%)	Switch, n (%)	Add-on, n (%)
Allopurinol	Combined	893	395 (44.2)	459 (51.4)	39 (4.4)
	BMC	312	138 (44.2)	163 (52.2)	11 (3.5)
	UUH	581	257 (44.2)	296 (51.0)	28 (4.8)
Febuxostat	Combined	1140	1036 (90.9)	95 (8.3)	9 (0.8)
	BMC	665	587 (88.3)	71 (10.7)	7 (1.10)
	UUH	475	449 (94.5)	24 (5.1)	2 (0.4)
Benzbromarone	Combined	187	80 (42.8)	106 (56.7)	1 (0.5)
	BMC	40	20 (50.0)	20 (50.0)	0 (0.0)
	UUH	147	60 (40.8)	86 (58.5)	1 (0.7)

BMC, Boramae Medical Center; UUH, Ulsan University Hospital.

^a^The data for patients who received combination therapy as first-line treatment were not displayed in the table due to the very low numbers.

Among allopurinol initiators who modified their initial treatment, retention rates numerically similar between those who switched to another agent and those who received add-on therapy (91.5% *vs*. 86.8%, *P* = 0.33; n = 459 *vs.* 39). Among febuxostat initiators, retention rates were 95.8% in switchers and 88.9% in add-on users (*P* = 0.36; n = 95 *vs.* 9). Among benzbromarone initiators, retention rates were 90.5% in switchers and 100% in add-on users (*P* = 1.00; n = 106 *vs*. 1).

### Subgroup analysis

In patients with CKD (n = 147), febuxostat was the predominant first-line ULT (72.1%), and 96.2% of patients continued therapy. Among CKD patients who initiated allopurinol, 48.6% continued treatment, while the remaining patients predominantly switched to another agent rather than adding an additional one (43.2% *vs*. 8.1%, *P* = 0.003).

**Fig 2:**
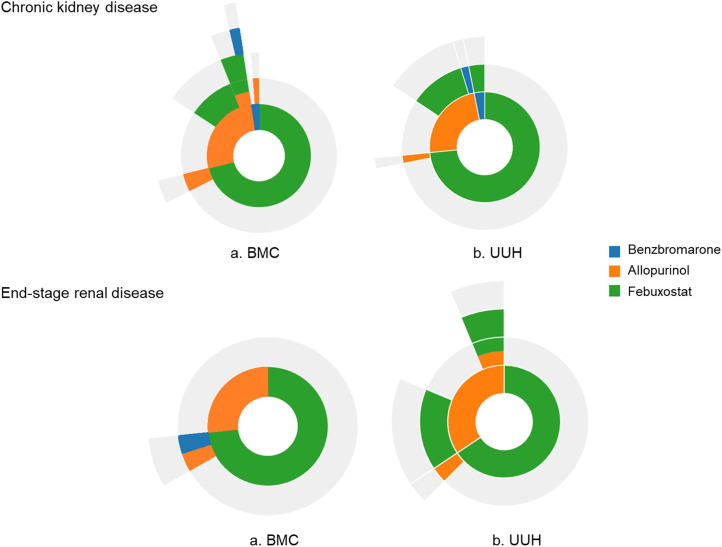
Sunburst plot showing treatment pathways of urate-lowering therapy in gout patients with chronic kidney disease or end-stage renal disease from each database. **(a)** Boramae Medical Center (BMC) and **(b)** Ulsan University Hospital (UUH).

In patients with ESRD (n = 62), febuxostat was also the most frequently used first-line agent (69.4%), with a retention rate of 93%. Among those who initiated allopurinol, approximately 63% continued treatment. Overall, patients with ESRD exhibited low variability in treatment sequences, similar to the patterns observed in CKD patients (Fig 2, S2 and S3 Tables in S1 File).

### Sensitivity analysis

Sequential treatment patterns were similar among patients who were continuously exposed to ULT during every 120-day interval (S1 Fig and S4 Table in S1 File). Febuxostat remained the most commonly used first-line agent (45.7%), with a retention rate of 88%. Approximately 60% of patients who initiated allopurinol or benzbromarone modified their treatment regimen. Among patients who did not achieve target serum urate levels of < 6 mg/dL for 6–12 months, the overall treatment pattern was consistent with that observed in the primary analysis (S2 Fig and S5 Table in S1 File). Analyses performed using the R-based method yielded results consistent with those obtained from the ATLAS Cohort Pathways analysis (S3 Fig in S1 File.).

To assess the impact of the index date definition on treatment sequence patterns, we also generated cohorts using either the first recorded gout diagnosis or the first ULT initiation date. Sunburst plots for both cohorts showed nearly identical patterns (data not shown), indicating that the choice of index date did not substantially affect the observed treatment sequences.

## Discussion

In this multicenter cohort of patients with gout, febuxostat was the most commonly prescribed first-line urate-lowering agent. The majority of patients maintained this treatment without any changes. However, 56% of patients who initially received allopurinol eventually changed their therapy, with most of them switching to febuxostat or benzbromarone rather than adding another agent. Among those who started with benzbromarone, 57.2% changed their therapy, with the majority also switching to a different agent. Overall, only a small number of patients progressed to third-line therapy, regardless of whether they switched or added on to their initial treatment. Patients with CKD or ESRD exhibited similar patterns in ULT sequences but were more likely to start with febuxostat as their initial treatment with low variability.

Current international guidelines emphasize a treat-to-target strategy, preferential use of XOIs, and switching to an alternative XOI rather than directly adding a uricosuric agent when initial therapy is inadequate. In this context, our findings suggest that real-world prescribing patterns in Korea are broadly consistent with guideline recommendations. The predominance of XOI monotherapy as initial therapy and the higher frequency of switching compared with add-on therapy reflect a step-wise escalation strategy. The very low proportion of patients initiating combination therapy is also consistent with cautious, guideline-concordant practice.

Although XOIs are widely used across countries, the relative proportions of allopurinol and febuxostat use differ substantially by region [16]. A U.S. claims data study reported that allopurinol accounted for nearly 90% of ULT prescriptions between 2009 and 2019, while febuxostat use declined after cardiovascular safety warnings [17]. In contrast, Japanese data have shown a more balanced use of febuxostat and allopurinol [18]. These regional differences may reflect variations in approved dosage ranges as well as population characteristics, including the higher prevalence of HLA-B*58:01 in Asian populations, which is associated with an increased risk of allopurinol hypersensitivity [18,19].

In Korea, a nationwide claims study from 2015 reported that 82% of ULT prescriptions were for allopurinol and 14% for febuxostat [20]. In contrast, our study, which analyzed data from 2010 to 2022 in secondary and tertiary hospitals in Korea, found that febuxostat was prescribed as the first-line therapy in 50% of patients, compared with 40% for allopurinol. This difference may partly reflect changes in prescribing patterns over time as well as differences in patient characteristics, given that referral centers are more likely to manage patients with comorbidities, such as CKD. Consistent with this interpretation, previous studies have reported that febuxostat users tended to have a higher burden of comorbidities and greater healthcare utilization [21,22].

Patients who initiated allopurinol were more likely to switch to another urate-lowering agent than to add an additional medication, but those who switched showed retention rates comparable to those receiving combination therapy. The 2020 American College of Rheumatology guidelines conditionally recommend switching to another XOI rather than adding a uricosuric agent when the response to initial XOI monotherapy is inadequate [3]. Pharmacoeconomic studies from Italy and Spain have demonstrated that the combination of lesinurad and allopurinol is more cost-effective as a second-line therapy than febuxostat monotherapy [23,24]. However, combination therapy may increase medication burden and reduce adherence [25], and the cost of febuxostat varies across countries. Therefore, switching to febuxostat appears to be a feasible option for patients who fail to achieve target serum urate levels with initial allopurinol monotherapy. Given that no significant difference in retention rates was observed between switchers and those on combination therapy in our study, combination therapy may be a reasonable alternative in settings where the cost of febuxostat is prohibitive.

In our study, more than 90% of patients who initiated febuxostat remained on treatment without modification, demonstrating a high retention rate compared with other ULT agents. This finding suggests substantial treatment stability in routine clinical practice. Among the minority who required changes, switching to another agent was more common than adding a medication, with comparable retention rates between the two groups. The most frequent modifications at BMC involved switching to allopurinol (56.4%, n = 44) or benzbromarone (34.6%, n = 27), followed by combination therapy with febuxostat and allopurinol (5.1%, n = 4) or febuxostat and benzbromarone (3.8%, n = 3). A similar pattern was observed among patients who failed to achieve the target serum urate level within 6–12 months (54% switched to allopurinol, 40% to benzbromarone, 4% to febuxostat plus allopurinol, and 2% to febuxostat plus benzbromarone). The particularly high retention observed with febuxostat may reflect several factors, including favorable tolerability, ease of dosing without extensive titration, and effectiveness in achieving target serum urate levels, especially in patients with renal impairment. In addition, concerns about allopurinol hypersensitivity in Asian populations may contribute to sustained use once febuxostat is selected as first-line therapy. Although causal inference cannot be established in this retrospective analysis, the consistently high retention rate underscores the clinical relevance of febuxostat as a stable first-line option in real-world practice.

Few studies have examined ULT patterns among patients with ESRD or those receiving dialysis. In our cohort, febuxostat was the most common first-line therapy for patients with ESRD (69%) and had a high retention rate (93%), whereas 63% of allopurinol initiators maintained therapy. Compared with the overall cohort, patients with ESRD had fewer treatment modifications and higher retention. This pattern may reflect the limited therapeutic options in severe renal impairment, given the contraindication of uricosuric agents [25], as well as safety considerations. Both Asian ethnicity and CKD are recognized risk factors for allopurinol hypersensitivity, which may influence clinicians to preferentially prescribe febuxostat in this population. Although international guidelines emphasize treat-to-target strategies and preferential XOI use, specific recommendations regarding ULT sequencing in advanced CKD or ESRD remain limited. Our findings therefore provide real-world insight into treatment selection and modification in this clinically complex group.

While our analysis focused on differences according to CKD and ESRD status, further stratified evaluations by other clinical characteristics, such as age, sex, comorbidity burden, or concomitant medications, may offer additional insight into prescribing decisions. Future studies are warranted to identify clinical factors associated with initial agent selection and subsequent treatment modification, including switching or add-on therapy, among patients receiving ULT.

To our knowledge, this is the first large-scale multicenter observational study to evaluate ULT sequences in patients with gout using longitudinal EHR data. The use of a CDM enabled standardized coding across hospitals, facilitating cross-institutional analysis. In addition, the large number of febuxostat initiators in our cohort allowed us to comprehensively examine subsequent treatment choices and their impact on therapy retention, providing valuable real-world insights into optimal treatment sequencing after febuxostat initiation.

Nevertheless, this study has several limitations. First, the reasons for switching or adding a new agent could not be directly identified because free-text clinical notes were not available in the CDM database. Therefore, we could not determine whether treatment modifications were driven by inadequate efficacy, adverse events, intolerance, cost, or patient preference. This limitation restricts the interpretation of the clinical intent behind treatment changes and precludes causal inference about the drivers of modification. Although sensitivity analyses restricted to patients who did not achieve target serum urate levels showed similar patterns, suggesting that inadequate efficacy may have contributed, this inference remains indirect. Furthermore, as this was a retrospective observational study, treatment allocation was not random. Differences in retention between drug groups cannot be attributed solely to differences in drug efficacy and should be interpreted in the context of baseline patient characteristics, physician preference, and other clinical factors that may have influenced treatment selection and sequencing. Accordingly, our findings should be understood as describing real-world treatment sequences rather than the specific causes of treatment decisions or comparative effectiveness between agents. Second, the data were derived exclusively from secondary and tertiary hospitals, which may limit the generalizability of our findings to patients managed in primary care settings. Patients treated at secondary and tertiary hospitals may have greater disease severity and comorbidity burden, which could influence initial drug selection and partly explain the higher proportion of febuxostat use as first-line therapy observed in this study. Nevertheless, the participating institutions include rheumatology specialists who are well versed in treat-to-target strategies and routinely implement treatment modifications based on serum urate levels and clinical response. Therefore, while the findings may not fully represent prescribing patterns in primary care, they likely reflect specialist-driven gout management with strong internal clinical validity. Third, the identification of gout and comorbidities was based on concept IDs, which may have introduced misclassification bias. Fourth, retention was defined as the absence of switching or add-on therapy and did not explicitly include treatment discontinuation, differing from conventional pharmacoepidemiologic definitions of drug persistence. The treatment pathway framework implemented through ATLAS Cohort Pathways and the TreatmentPatterns package focuses on transitions between treatment eras and does not classify discontinuation as a separate state. Consequently, patients who discontinued ULT without initiating alternative therapy may have been misclassified as retained, potentially overestimating traditional persistence. In sensitivity analyses restricted to patients with continuous ULT exposure at least every 120 days, the findings were consistent. Accordingly, retention in this study should be interpreted as reflecting stability within treatment sequences rather than conventional drug persistence. Finally, our hospital-based CDM data do not capture prior ULT use at other healthcare institutions. As a result, some patients classified as first-line initiators may have been prevalent users, potentially misclassifying the true index therapy. In such cases, a drug recorded as the initial treatment may in fact represent a switched therapy, which may alter the observed sequence of treatment transitions.

In summary, our real-world data indicate that ULT is typically used as first- or second-line therapy in patients with gout, with relatively few requiring third-line treatment. Febuxostat was the most frequently initiated agent and showed high retention, whereas allopurinol and benzbromarone were more often modified after initiation. Switching was more common than add-on therapy, although retention rates were similar between strategies. These patterns may be particularly relevant in Asian populations, where the higher prevalence of HLA-B*58:01 and concerns about allopurinol hypersensitivity may influence treatment selection. In clinical practice, decisions about initial agent choice and subsequent modification should be individualized, taking into account adherence, comorbidities, safety considerations, and cost. Our results provide insight into how ULT agents are initiated and sequenced in routine specialist care, particularly in Asian populations.

## Supporting information

S1 File S1 Fig. Sunburst plot showing treatment pathways in patients with continuous exposure to the urate-lowering agents in every 120-day period. (a) Boramae Medical Center (BMC) and (b) Ulsan University Hospital (UUH). **S2 Fig.** Sunburst plot showing treatment pathways in patients whose serum urate levels remained at 6 mg/dL or higher between 6 and 12 months after the index date. (a) Boramae Medical Center (BMC) and (b) Ulsan University Hospital (UUH). **S3 Fig.** Sunburst plot showing treatment pathways analyzed using the R package TreatmentPatterns. (a) Boramae Medical Center (BMC) and (b) Ulsan University Hospital (UUH). **S1 Table in S1 File.** Concept ID information. **S2 Table in S1 File.** Sequential treatment patterns of urate-lowering therapy in gout patients with chronic kidney disease. **S3 Table in S1 File.** Sequential treatment patterns of urate-lowering therapy in gout patients with end-stage renal disease. **S4 Table in S1 File.** Sequential treatment pattern of urate-lowering therapy in patients with continuous exposure in every 120-day period. **S5 Table in S1 File.** Sequential treatment patterns of urate-lowering therapy in gout patients who failed to reach the target serum urate between 6 and 12 months after the index date. **S6 Table in S1 File.** Detailed treatment pathway frequency by institution.
(ZIP)
