# Supplementary material for: Treatment sequence patterns of urate-lowering therapy in Korean patients with gout: A common data model-based study
Source: PLoS One. 2026 Apr 17;21(4):e0347654. doi: 10.1371/journal.pone.0347654 (PMC13089728; doi:10.1371/journal.pone.0347654)
Supplement: S1 File — Sunburst plot showing treatment pathways in patients with continuous exposure to the urate-lowering agents in every 120-day period. (a) Boramae Medical Center (BMC) and (b) Ulsan University Hospital (UUH). S2 Fig. Sunburst plot showing treatment pathways in patients whose serum urate levels remained at 6 mg/dL or higher between 6 and 12 months after the index date. (a) Boramae Medical Center (BMC) and (b) Ulsan University Hospital (UUH). S3 Fig. Sunburst plot showing treatment pathways analyzed using the R package TreatmentPatterns. (a) Boramae Medical Center (BMC) and (b) Ulsan University Hospital (UUH). S1 Table in S1 File. Concept ID information. S2 Table in S1 File. Sequential treatment patterns of urate-lowering therapy in gout patients with chronic kidney disease. S3 Table in S1 File. Sequential treatment patterns of urate-lowering therapy in gout patients with end-stage renal disease. S4 Table in S1 File. Sequential treatment pattern of urate-lowering therapy in patients with continuous exposure in every 120-day period. S5 Table in S1 File. Sequential treatment patterns of urate-lowering therapy in gout patients who failed to reach the target serum urate between 6 and 12 months after the index date. S6 Table in S1 File. Detailed treatment pathway frequency by institution. (ZIP) [file pone.0347654.s001.zip › Supporting information/S1 Table.pdf]

S1 Table. Concept ID information

Concept set	Concept name	Concept ID	Vocabulary
Gout	Gouty tophus	4299408	SNOMED
	Chronic gouty arthritis	4285308	SNOMED
	Primary gout	4084229	SNOMED
	Gout secondary to drug	4035751	SNOMED
	Gout secondary to renal impairment	4035437	SNOMED
	Secondary gout	4035436	SNOMED
	Gout	440674	SNOMED
	Articular gout	74892	SNOMED
CKD	Chronic kidney disease stage 3	443597	SNOMED
	Chronic kidney disease stage 4	443612	SNOMED
ESRD	End-stage renal disease	193782	SNOMED
	End stage renal failure on dialysis	4030520	SNOMED
	Chronic kidney disease stage 5	443611	SNOMED
Allopurinol	Allopurinol 100 MG Oral Tablet	1167323	RxNorm
	Allopurinol	1167322	RxNorm
	Allopurinol 100 MG	19080868	RxNorm
Febuxostat	Febuxostat 80 MG Oral Tablet	19017747	RxNorm
	Febuxostat 40 MG Oral Tablet	19017744	RxNorm
	Febuxostat	19017742	RxNorm
	Febuxostat 40 MG	19017743	RxNorm
	Febuxostat 80 MG	19133952	RxNorm
Benzbromarone	Benzbromarone 50 MG Oral Tablet	19107469	RxNorm
	Benzbromarone	19016754	RxNorm
Serum urate	Urate in serum or plasma	3037556	LOINC

CKD, chronic kidney disease; ESRD, end-stage renal disease; LOINC, Logical Observation Identifiers Names and Codes; SNOMED, Systematized Nomenclature of Medicine
