# Supplementary material for: Treatment sequence patterns of urate-lowering therapy in Korean patients with gout: A common data model-based study
Source: PLoS One. 2026 Apr 17;21(4):e0347654. doi: 10.1371/journal.pone.0347654 (PMC13089728; doi:10.1371/journal.pone.0347654)
Supplement: S1 File — Sunburst plot showing treatment pathways in patients with continuous exposure to the urate-lowering agents in every 120-day period. (a) Boramae Medical Center (BMC) and (b) Ulsan University Hospital (UUH). S2 Fig. Sunburst plot showing treatment pathways in patients whose serum urate levels remained at 6 mg/dL or higher between 6 and 12 months after the index date. (a) Boramae Medical Center (BMC) and (b) Ulsan University Hospital (UUH). S3 Fig. Sunburst plot showing treatment pathways analyzed using the R package TreatmentPatterns. (a) Boramae Medical Center (BMC) and (b) Ulsan University Hospital (UUH). S1 Table in S1 File. Concept ID information. S2 Table in S1 File. Sequential treatment patterns of urate-lowering therapy in gout patients with chronic kidney disease. S3 Table in S1 File. Sequential treatment patterns of urate-lowering therapy in gout patients with end-stage renal disease. S4 Table in S1 File. Sequential treatment pattern of urate-lowering therapy in patients with continuous exposure in every 120-day period. S5 Table in S1 File. Sequential treatment patterns of urate-lowering therapy in gout patients who failed to reach the target serum urate between 6 and 12 months after the index date. S6 Table in S1 File. Detailed treatment pathway frequency by institution. (ZIP) [file pone.0347654.s001.zip › Supporting information/S5 Table.pdf]

S5 Table. Sequential treatment patterns of urate-lowering therapy in gout patients who failed to reach the target serum urate between 6 and 12 months after the index date

Initial therapy[a]	Cohort	n	Continuation, n (%)	Switch, n (%)	Add-on, n (%)
Allopurinol	Combined	435	186 (42.8)	233 (53.6)	16 (3.7)
	BMC	146	62 (42.5)	78 (53.4)	6 (4.1)
	UUH	289	124 (42.9)	155 (53.6)	10 (3.5)
Febuxostat	Combined	450	387 (86.0)	60 (13.3)	3 (0.7)
	BMC	274	224 (81.8)	47 (17.2)	3 (1.1)
	UUH	176	163 (92.6)	13 (7.4)	0 (0)
Benzbromarone	Combined	74	25 (33.8)	48 (64.9)	1 (1.4)
	BMC	15	10 (66.7)	5 (33.3)	0 (0)
	UUH	59	15 (25.4)	43 (72.9)	1 (1.7)

BMC, Boramae Medical Center; UUH, Ulsan University Hospital.

[a]The data for patients who received combination therapy as first-line treatment were not displayed in the table due to the very low numbers.
