# Supplementary material for: Treatment sequence patterns of urate-lowering therapy in Korean patients with gout: A common data model-based study
Source: PLoS One. 2026 Apr 17;21(4):e0347654. doi: 10.1371/journal.pone.0347654 (PMC13089728; doi:10.1371/journal.pone.0347654)
Supplement: S1 File — Sunburst plot showing treatment pathways in patients with continuous exposure to the urate-lowering agents in every 120-day period. (a) Boramae Medical Center (BMC) and (b) Ulsan University Hospital (UUH). S2 Fig. Sunburst plot showing treatment pathways in patients whose serum urate levels remained at 6 mg/dL or higher between 6 and 12 months after the index date. (a) Boramae Medical Center (BMC) and (b) Ulsan University Hospital (UUH). S3 Fig. Sunburst plot showing treatment pathways analyzed using the R package TreatmentPatterns. (a) Boramae Medical Center (BMC) and (b) Ulsan University Hospital (UUH). S1 Table in S1 File. Concept ID information. S2 Table in S1 File. Sequential treatment patterns of urate-lowering therapy in gout patients with chronic kidney disease. S3 Table in S1 File. Sequential treatment patterns of urate-lowering therapy in gout patients with end-stage renal disease. S4 Table in S1 File. Sequential treatment pattern of urate-lowering therapy in patients with continuous exposure in every 120-day period. S5 Table in S1 File. Sequential treatment patterns of urate-lowering therapy in gout patients who failed to reach the target serum urate between 6 and 12 months after the index date. S6 Table in S1 File. Detailed treatment pathway frequency by institution. (ZIP) [file pone.0347654.s001.zip › Supporting information/S6 Table.pdf]

S6 Table. Detailed treatment pathway frequency by institution

Treatment pathway	BMC (n=1019)	UUH (n=1213)
Febuxostat-End	587 (57.61)	449 (39.02)
Allopurinol-febuxostat-End	157 (15.41)	252 (20.77)
Allopurinol-End	138 (13.54)	257 (21.19)
Febuxostat-allopurinol-End	42 (4.12)	18 (1.48)
Febuxostat-benzbromarone-End	25 (2.45)	6 (0.49)
Benzbromarone-End	20 (1.96)	60 (4.95)
Benzbromarone-febuxostat-End	15 (1.47)	72 (5.94)
Allopurinol-allopurinol+febuxostat-febuxostat-End	8 (0.79)	23 (1.90)
Allopurinol-benzbromarone-febuxostat-End	0 (0)	23 (1.90)
Allopurinol-benzbromarone-End	0 (0)	11 (0.91)
Allopurinol-febuxostat-benzbromarone-End	6 (0.59)	9 (0.74)
Benzbromarone-allopurinol-End	4 (0.39)	4 (0.33)
Febuxostat-allopurinol+febuxostat-allopurinol-End	3 (0.29)	0 (0)
Febuxostat-allopurinol-benzbromarone-End	2 (0.20)	0 (0)
Febuxostat-benzbromarone-allopurinol-End	2 (0.20)	0 (0)
Febuxostat-benzbromarone+febuxostat-End	2 (0.20)	1 (0.08)
Allopurinol-allopurinol+febuxostat-febuxostat-benzbromarone-End	2 (0.20)	1 (0.08)
Benzbromarone-allopurinol-febuxostat-End	1 (0.10)	6 (0.49)
Benzbromarone-febuxostat-allopurinol-End	0 (0)	4 (0.33)
Benzbromarone+febuxostat-End	1 (0.10)	0 (0)
Allopurinol-allopurinol+benzbromarone-febuxostat-End	0 (0)	2 (0.16)
Allopurinol-allopurinol+febuxostat-End	1 (0.10)	0 (0)
Benzbromarone-febuxostat-End	1 (0.10)	0 (0)
Febuxostat-benzbromarone+febuxostat-benzbromarone-allopurinol-End	1 (0.10)	0 (0)
Febuxostat-febuxostat+allopurinol	1 (0.10)	1 (0.08)
Benzbromarone+allopurinol-End	0 (0)	8 (0.66)
Benzbromarone+allopurinol-allopurinol-End	0 (0)	1 (0.08)
Benzbromarone+allopurinol-allopurinol-febuxostat-End	0 (0)	1 (0.08)
Allopurinol-benzbromarone+febuxostat	0 (0)	1 (0.08)
Benzbromarone-benzbromarone+febuxostat	0 (0)	1 (0.08)
Allopurinol-benzbromarone+allopurinol-benzbromarone-End	0(0)	1 (0.08)

BMC, Boramae Medical Center; UUH, Ulsan University Hospital.
